# Derivation and External Validation of a Risk Index for Predicting Acute Kidney Injury Requiring Kidney Replacement Therapy After Noncardiac Surgery

**DOI:** 10.1001/jamanetworkopen.2021.21901

**Published:** 2021-08-23

**Authors:** Todd A. Wilson, Lawrence de Koning, Robert R. Quinn, Kelly B. Zarnke, Eric McArthur, Carina Iskander, Pavel S. Roshanov, Amit X. Garg, Brenda R. Hemmelgarn, Neesh Pannu, Matthew T. James

**Affiliations:** 1 Department of Community Health Sciences, Cumming School of Medicine, University of Calgary, Calgary, Alberta, Canada; 2 Department of Pathology and Laboratory Medicine, University of Calgary, Calgary, Alberta, Canada; 3 Alberta Precision Laboratories, Calgary, Alberta, Canada; 4 Libin Cardiovascular Institute, Cumming School of Medicine, University of Calgary, Calgary, Alberta, Canada; 5 Department of Medicine, Cumming School of Medicine, University of Calgary, Calgary, Alberta, Canada; 6 O’Brien Institute for Public Health, Cumming School of Medicine, University of Calgary, Calgary, Alberta, Canada; 7 ICES, Toronto, Ontario, Canada; 8 Department of Medicine, Western University, London, Ontario, Canada; 9 Department of Epidemiology & Biostatistics, Western University, London, Ontario, Canada; 10 Department of Medicine, University of Alberta, Edmonton, Alberta, Canada

## Abstract

**Importance:**

Severe acute kidney injury (AKI) is a serious postoperative complication. A tool for predicting the risk of AKI requiring kidney replacement therapy (KRT) after major noncardiac surgery might assist with patient counseling and targeted use of measures to reduce this risk.

**Objective:**

To derive and validate a predictive model for AKI requiring KRT after major noncardiac surgery.

**Design, Setting, and Participants:**

In this prognostic study, 5 risk prediction models were derived and internally validated in a population-based cohort of adults without preexisting kidney failure who underwent noncardiac surgery in Alberta, Canada, between January 1, 2004, and December 31, 2013. The best performing model and corresponding risk index were externally validated in a population-based cohort of adults without preexisting kidney failure who underwent noncardiac surgery in Ontario, Canada, between January 1, 2007, and December 31, 2017. Data analysis was conducted from September 1, 2019, to May 31, 2021.

**Exposures:**

Demographic characteristics, surgery type, laboratory measures, and comorbidities before surgery.

**Main Outcomes and Measures:**

Acute kidney injury requiring KRT within 14 days after surgery. Discrimination was assessed using the *C* statistic; calibration was assessed using calibration intercept and slope. Logistic recalibration was used to optimize model calibration in the external validation cohort.

**Results:**

The derivation cohort included 92 114 patients (52.2% female; mean [SD] age, 62.3 [18.0] years), and the external validation cohort included 709 086 patients (50.8% female; mean [SD] age, 61.0 [16.0] years). A total of 529 patients (0.6%) developed postoperative AKI requiring KRT in the derivation cohort, and 2956 (0.4%) developed postoperative AKI requiring KRT in the external validation cohort. The following factors were consistently associated with the risk of AKI requiring KRT: younger age (40-69 years: odds ratio [OR], 2.07 [95% CI, 1.69-2.53]; <40 years: OR, 3.73 [95% CI, 2.61-5.33]), male sex (OR, 1.55; 95% CI, 1.28-1.87), surgery type (colorectal: OR, 4.86 [95% CI, 3.28-7.18]; liver or pancreatic: OR, 6.46 [95% CI, 3.85-10.83]; other abdominal: OR, 2.19 [95% CI, 1.66-2.89]; abdominal aortic aneurysm repair: OR, 19.34 [95% CI, 14.31-26.14]; other vascular: OR, 7.30 [95% CI, 5.48-9.73]; thoracic: OR, 3.41 [95% CI, 2.07-5.59]), lower estimated glomerular filtration rate (OR, 0.97; 95% CI, 0.97-0.97 per 1 mL/min/1.73 m^2^ increase), lower hemoglobin concentration (OR, 0.99; 95% CI, 0.98-0.99 per 0.1 g/dL increase), albuminuria (mild: OR, 1.88 [95% CI, 1.52-2.33]; heavy: OR, 3.74 [95% CI, 2.98-4.69]), history of myocardial infarction (OR, 1.63; 95% CI, 1.32-2.03), and liver disease (mild: OR, 2.32 [95% CI, 1.66-3.24]; moderate or severe: OR, 4.96 [95% CI, 3.58-6.85]). In external validation, a final model including these variables showed excellent discrimination (*C* statistic, 0.95; 95% CI, 0.95-0.96), with sensitivity of 21.2%, specificity of 99.9%, positive predictive value of 38.1%, and negative predictive value of 99.7% at a predicted risk threshold of 10% or greater.

**Conclusions and Relevance:**

The findings suggest that this risk model can predict AKI requiring KRT after noncardiac surgery using routine preoperative data. The model may be feasible for implementation in clinical perioperative risk stratification for severe AKI.

## Key Points

**Question:**

Can acute kidney injury requiring kidney replacement therapy after major noncardiac surgery be accurately predicted from readily available preoperative data?

**Findings:**

In this prognostic study of a risk prediction model developed with 92 114 adults and externally validated with 709 086 adults, in external validation, a model including age, sex, surgery type, estimated glomerular filtration rate, hemoglobin concentration, albuminuria, and history of myocardial infarction or liver disease had sensitivity of 21.2%, specificity of 99.9%, positive predictive value of 38.1%, and negative predictive value of 99.7% at a predicted risk threshold of 10% or greater.

**Meaning:**

The findings suggest that this risk model can predict acute kidney injury requiring kidney replacement therapy after noncardiac surgery using routine preoperative data and may be feasible for implementation in perioperative care.

## Teaser

This prognostic study derives and validates a predictive model for acute kidney injury requiring kidney replacement therapy after major noncardiac surgery.

## Introduction

Acute kidney injury (AKI) is common after major surgery and is associated with adverse outcomes, including prolonged hospitalization, chronic kidney disease, and death.^1,2,3^ Postoperative AKI accounts for more than one-third of cases of AKI in the hospital setting,^4^ and the incidence of AKI requiring kidney replacement therapy (KRT) after major elective surgery increased almost 4-fold between 1995 and 2009.^5,6^ When AKI is severe enough to require KRT, it is resource intensive and is associated with adverse patient experiences and outcomes.^7,8^ Quantifying the risk of AKI requiring KRT is an important part of the surgical informed consent process and may impact perioperative decision-making.^9,10^

Several validated prediction models for AKI after cardiac surgery are available; however, few such tools exist for AKI after noncardiac surgical procedures,^11,12^ even though the risk may be substantial for some patients.^5,6,13,14^ Clinical practice guidelines^15^ have included the recommendation that patients be stratified for risk of AKI. However, a lack of accurate risk-stratification approaches for clinically significant AKI after noncardiac surgery is a barrier to further research and clinical uptake of approaches to mitigate the risk of AKI. Accurate identification of patients at increased risk for AKI may allow for increased monitoring and supportive strategies. Therefore, AKI guidelines from Kidney Disease: Improving Global Outcomes have included a recommendation for research in the development and validation of AKI risk prediction tools for use in contexts other than cardiac surgery.^15^

We used population-based cohorts to develop and externally validate risk prediction models and an integer-based risk index for AKI requiring KRT after major noncardiac surgery. We framed the risk prediction tools for risk stratification in preoperative clinical settings and focused on risk factors readily identifiable before surgery.

## Methods

This prognostic study was approved by the Conjoint Health Research Ethics Board at the University of Calgary, Calgary, Alberta, Canada, with a waiver of informed consent because retrospective deidentified data were used. We adhered to the Transparent Reporting of a Multivariable Prediction Model for Individual Prognosis or Diagnosis (TRIPOD) reporting guideline for reporting multivariable prediction model development and validation.^16^

### Derivation and Validation Cohorts

We obtained the model derivation cohort from the Alberta Kidney Disease Network database and Alberta Health administrative data using approaches described elsewhere.^17^ We used *International Classification of Diseases, Ninth Revision, Clinical Modification* (*ICD-9-CM*) from physician claims in Alberta and *International Statistical Classification of Diseases and Related Health Problems, Tenth Revision (ICD-10)* and *Canadian Classification of Health Intervention* procedure codes from hospital discharge abstracts to characterize patient comorbidities and procedures.^18,19,20,21^ We included all patients aged 18 to 95 years who underwent surgical repair of abdominal aortic aneurysm or had surgical procedures performed as treatment for colorectal, liver, or pancreatic disease and those who underwent other abdominal, retroperitoneal, thoracic, vascular, or musculoskeletal surgical procedures in Alberta, Canada, between January 1, 2004, and December 31, 2013. We excluded patients without a preoperative serum creatinine measurement within 30 days before surgery and patients with end-stage kidney disease (defined by receipt of dialysis or kidney transplant or a baseline estimated glomerular filtration rate [eGFR] <10 mL/min/1.73 m^2^) before surgery. Diagnostic and procedure codes for cohort development and characterization are shown in eTables 1 and 2 in the Supplement.

The external validation cohort was derived using data sets from Ontario, Canada, from January 1, 2007, through December 31, 2017, that were analyzed at ICES (formerly Institute for Clinical Evaluative Science) in London, Ontario. ICES houses laboratory information and provincial administrative data for patients in Ontario, Canada. Data analysis was conducted from September 1, 2019, to May 31, 2021. Cohort inclusion criteria were identical to those of the derivation cohort, with similar *ICD-9-CM* and *ICD-10* coding approaches used to characterize patient comorbidities and procedures.^22,23^

### Outcome Measure and Candidate Predictor Variables

We defined AKI requiring KRT using *ICD-10* diagnosis codes for AKI and *Canadian Classification of Health* procedure codes for KRT (eTable 1 in the Supplement) within 14 days of surgery by following a validated approach that has been shown to have high sensitivity and specificity for AKI requiring KRT.^20,21,24^ We identified candidate predictor variables by means of literature review and their routine availability in clinical care.^17,25,26,27^ We included demographic data, laboratory measures, comorbidities, surgical procedures,^5^ and status of the surgery as emergent or urgent vs elective. We used the closest measurement within 30 days and included the patient’s date of surgery to determine the baseline serum creatinine concentration and eGFR based on the Chronic Kidney Disease Epidemiology Collaboration (CKD-EPI) equation.^28^ We used hemoglobin measurements from before surgery closest to the patient’s surgery date. Albuminuria was based on measurements closest to the surgery date using urine dipstick or the albumin to creatinine ratio, categorized as normal (negative; <30 mg/g; to convert albumin to creatine ratio to mg/mmol, multiply by 0.113), mild (trace; ≥1 or 30-300 mg/g), or heavy (≥2 or ≥300 mg/g) within 1 year before surgery.^29^ We identified comorbidities up to 3 years before surgery from hospital discharge abstracts and physician claims using validated coding algorithms.^18,19^

### Statistical Analysis

We evaluated the linearity of associations between the outcome and continuous variables (age, eGFR, and hemoglobin concentration) using locally weighted scatterplot smoothing and restricted cubic spline analysis. We confirmed linear associations for eGFR and hemoglobin concentration with AKI requiring KRT. Locally weighted scatterplot smoothing analysis showed a quadratic association of the outcome with age, which we therefore categorized as younger than 40 years, 40 to 69 years, and 70 years or older in the models. To address unmeasured albuminuria status, we performed a single imputation, separately within each cohort, with an ordinal logistic regression model with all variables included in the full model to impute missing values. Missing hemoglobin measurements for 22 patients (<0.001%) were imputed to the mean hemoglobin concentration in the cohort (127 g/L; to convert to g/dL, divide by 10.0).

We developed 5 multivariable logistic regression models based on 3 different strategies. First, we fit a full model including all candidate predictors. We performed bootstrap resampling for variable selection using 1000 bootstrap samples of the full cohort and fit a model using the variables that were statistically significant at a 2-sided 1% level of significance in at least 80% of bootstrap samples. We developed other models with increasing simplicity by further removing variables that might be less consistently available in a preoperative setting. We determined the discrimination of each model in the derivation cohort by calculating the apparent *C* statistic.

The full model (model 1) included 30 variables. After bootstrap resampling, 15 variables were selected in at least 80% of samples and included in model 2. Model 3 included variables from model 2 but excluded albuminuria. Model 4 consisted of age, sex, surgery type, eGFR, and hemoglobin concentration (10 variables); only age, sex, and surgery type were included in model 5.

We internally validated each model using bootstrap resampling. We took 100 bootstrap samples and fit the respective model, calculated the *C* statistic, and determined bootstrap and test *C* statistics. We subtracted the difference between the bootstrap statistic and test *C* statistic from the apparent *C* statistic to determine the optimism-corrected *C* statistic for each model.^30^

We evaluated calibration based on the calibration intercept and slope from the 100 bootstrap samples using logistic regression of the predicted risk against the outcome. The average of the 100 regression coefficients was taken as the calibration slope for the respective model. Perfect calibration is indicated by a calibration intercept of 0 and calibration slope of 1.^30,31^

We further compared models using the bayesian information criteria, area under the precision recall curve, categorical net reclassification improvement, and integrated discrimination improvement. The net reclassification improvement assesses the improvement in stratification of cases and controls into high and low predicted risk categories with the addition of new variables.^32,33^ We prespecified risk categories of less than 1%, 1% to less than 5%, 5% to less than 10%, 10% to less than 20%, and 20% or greater based on clinician input. The integrated discrimination improvement is the difference between mean predicted risks of patients with events and nonevents between the new and old models.^32,34,35^ We further compared models using reclassification tables with stratification of events and nonevents and the same prespecified predicted risk categories.^36^

We transformed the best performing model based on accuracy and parsimony into an integer-based risk index using methods from the Framingham Heart Study.^37^ This process assigns an integer number of points to each variable, and the total points for each patient in the cohort are used to estimate the corresponding risk.

We performed external validation of the best performing model and corresponding risk index by applying them to the external validation cohort from ICES Ontario to evaluate their predictive performance in a geographically distinct, independent cohort. We assessed discrimination with the *C* statistic and calibration with calibration intercept and slope. We used logistic recalibration to optimize model calibration in the external validation cohort.^38^ Analyses were performed using Stata, version 16 (StataCorp LLC).

## Results

### Cohort Descriptions

**Figure 1.**
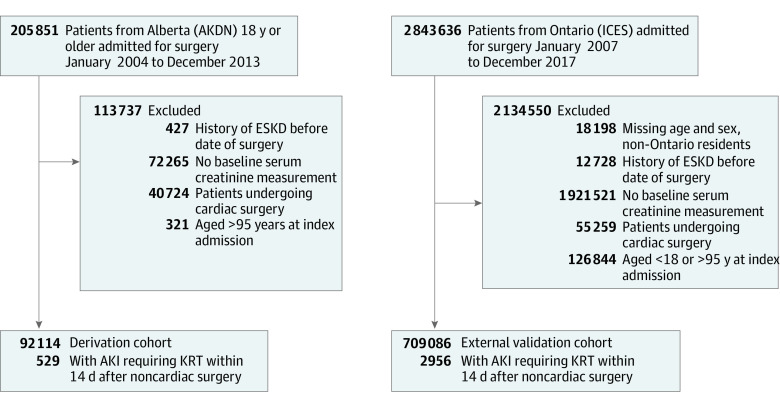
Flowcharts of the Derivation and External Validation Cohorts AKDN indicates Alberta Kidney Disease Network; AKI, acute kidney injury; ESKD, end-stage kidney disease; KRT, kidney replacement therapy.

The derivation cohort included 92 114 patients (47.8% male and 52.2% female; mean [SD] age, 62.3 [18.0] years); 529 (0.6%) developed AKI requiring KRT within 14 days of noncardiac surgery (Figure 1). Patients who developed AKI requiring KRT were more often male; had undergone repair of abdominal aortic aneurysm or vascular, colorectal, liver, or pancreatic surgery; and had lower baseline eGFR and hemoglobin concentration (eTable 3 in the Supplement).

The external validation cohort included 709 086 patients (49.2% male and 50.8% female; mean [SD] age, 61.0 [16.0] years); 2956 (0.4%) developed AKI requiring KRT within 14 days of noncardiac surgery. The mean age was similar to that of the derivation cohort; however, the percentages of patients younger than 40 years and 70 years or older were smaller in the external validation cohort (<40 years: 10.6% vs 13.3%; ≥70 years: 32.2% vs 38.9%) (Table 1). In the external validation cohort, a larger percentage of patients had moderate or severe liver disease, a history of abdominal and musculoskeletal surgery, and a higher mean baseline eGFR. A smaller percentage of patients had a history of myocardial infarction or abdominal aortic aneurysm and vascular, colorectal, liver, or pancreatic surgery.

**Table 1.** Baseline Characteristics of the Derivation and External Validation Cohorts

Characteristic	Individuals^a^	
	Derivation cohort (n = 92 114)	External validation cohort (n = 709 086)
Age, mean (SD), y	62.3 (18.0)	61.0 (16.0)
Age, y		
<40	12 221 (13.3)	75 285 (10.6)
40-69	44 027 (47.8)	44 027 (57.2)
≥70	35 866 (38.9)	228 225 (32.2)
Sex		
Male	44 034 (47.8)	349 013 (49.2)
Female	48 080 (52.2)	360 073 (50.8)
Emergent or urgent surgery	50 231 (54.5)	171 513 (24.2)
Surgery type		
Musculoskeletal	42 864 (46.5)	359 602 (50.7)
Colorectal	3585 (3.9)	6540 (0.9)
Liver or pancreatic	1371 (1.5)	3894 (0.6)
Other abdominal	30 346 (32.9)	273 600 (38.6)
AAA	2217 (2.4)	678 (0.1)
Other vascular	5036 (5.5)	19 295 (2.7)
Thoracic	3783 (4.1)	20 523 (2.9)
Retroperitoneal	2912 (3.2)	24 954 (3.5)
Comorbidities		
Cancer	25 397 (27.6)	218 334 (30.8)
Cerebrovascular disease	8957 (9.7)	39 983 (5.6)
Congestive heart failure	10 077 (10.9)	47 139 (6.6)
COPD	24 027 (26.1)	74 828 (10.6)
Dementia	6443 (7.0)	39 214 (5.5)
HIV infection or AIDS	163 (0.2)	1896 (0.3)
Metastatic carcinoma	8489 (9.2)	28 485 (4.0)
Myocardial infarction	8354 (9.1)	18 629 (2.6)
Mild liver disease^b^	3033 (3.3)	10 620 (1.5)
Moderate or severe liver disease^c^	1095 (1.2)	37 283 (5.3)
Paraplegia or hemiplegia	1724 (1.9)	4355 (0.6)
Peptic ulcer disease	5331 (5.8)	13 219 (1.9)
Peripheral vascular disease	10 587 (11.5)	15 837 (2.2)
Rheumatic or connective tissue disease	3702 (4.0)	7441 (1.0)
Diabetes	18 343 (19.9)	173 910 (24.5)
Hypertension	11 488 (12.5)	349 681 (49.3)
Preoperative laboratory measures		
eGFR, mean (SD), mL/min/1.73 m^2^	79.3 (24.8)	83.0 (22.4)
Hemoglobin concentration, mean (SD), g/dL	12.7 (2.2)	13.4. (1.8)
Albuminuria^d^		
Normal albumin level	49 427 (53.7)	201 105 (28.4)
Mild	13 545 (14.7)	33 118 (4.7)
Heavy	4833 (5.2)	12 611 (1.8)
Unmeasured	24 309 (26.4)	462 252 (65.2)
Albuminuria imputed^d^		
Normal albumin level	67 732 (73.5)	592 373 (83.5)
Mild	17 946 (19.5)	86 324 (12.2)
Heavy	6436 (7.0)	30 389 (4.3)

Abbreviations: AAA, abdominal aortic aneurysm; ACR, albumin to creatinine ratio; COPD, chronic obstructive pulmonary disease; eGFR, estimated glomerular filtration rate.

SI conversion factor: To convert hemoglobin concentration to grams per liter, multiply by 10.0; and urinary ACR to milligrams per millimole, multiply by 0.113.

^a^ Data are presented as number (percentage) of individuals unless otherwise indicated.

^b^ Mild liver disease includes chronic liver diseases and cirrhosis.

^c^ Moderate or severe liver diseases include esophageal varices with or without bleeding, hepatic encephalopathy, portal hypertension, hepatorenal syndrome, and other sequelae of chronic liver disease.

^d^ Normal albumin level was defined by dipstick urinalysis protein negative or an ACR of less than 30 mg/g; mild albuminuria, dipstick urinalysis protein trace or greater than or equal to 1 or ACR from 30 to 300 mg/g; and heavy, dipstick urinalysis protein of greater than or equal to 2 or ACR of more than 300 mg/g.

### Model Characteristics

With bootstrap resampling (model 2), the following factors were associated with the risk of AKI requiring KRT: younger age (40-69 years: odds ratio [OR], 2.07 [95% CI, 1.69-2.53]; <40 years: OR, 3.73 [95% CI, 2.61-5.33]), male sex (OR, 1.55; 95% CI, 1.28-1.87), surgery type (colorectal: OR, 4.86 [95% CI, 3.28-7.18]; liver or pancreatic: OR, 6.46 [95% CI, 3.85-10.83]; other abdominal: OR, 2.19 [95% CI, 1.66-2.89]; abdominal aortic aneurysm repair: OR, 19.34 [95% CI, 14.31-26.14]; other vascular: OR, 7.30 [95% CI, 5.48-9.73]; thoracic: OR, 3.41 [95% CI, 2.07-5.59]), lower eGFR (OR, 0.97; 95% CI, 0.97-0.97 per 1 mL/min/1.73 m^2^ increase), lower hemoglobin concentration (OR, 0.99; 95% CI, 0.98-0.99 per 0.1 g/dL increase), albuminuria (mild: OR, 1.88 [95% CI, 1.52-2.33]; heavy: OR, 3.74 [95% CI, 2.98-4.69]), history of myocardial infarction (OR, 1.63; 95% CI, 1.32-2.03), and liver disease (mild: OR, 2.32 [95% CI, 1.66-3.24]; moderate or severe: OR, 4.96 [95% CI, 3.58-6.85]). The predictive variables and odds ratios for the full model (model 1) and reduced models (models 3-5) are shown in Table 2.

**Table 2.** Odds Ratios for Predictive Variables Included in Each Model Developed in the Derivation Cohort^a^

Predictive variable	Odds ratio (95% CI)					
	Model 1	Model 2	Model 3	Model 4	Model 5
Age, y					
<40	3.15 (2.17-4.57)	3.73 (2.61-5.33)	4.58 (3.21-6.53)	4.32 (3.04-6.12)	0.89 (0.65-1.23)
40-69	1.94 (1.57-2.39)	2.07 (1.69-2.53)	2.23 (1.82-2.72)	2.42 (1.99-2.94)	1.18 (0.98-1.42)
≥70	1 [Reference]	1 [Reference]	1 [Reference]	1 [Reference]	1 [Reference]
Sex					
Male	1.52 (1.25-1.85)	1.55 (1.28-1.87)	1.73 (1.43-2.09)	1.83 (1.52-2.21)	1.48 (1.23-1.79)
Female	1 [Reference]	1 [Reference]	1 [Reference]	1 [Reference]	1 [Reference]
Surgery type					
Musculoskeletal	1 [Reference]	1 [Reference]	1 [Reference]	1 [Reference]	1 [Reference]
Colorectal	4.80 (3.19-7.23)	4.86 (3.28-7.18)	5.08 (3.44-7.50)	5.15 (3.50-7.60)	5.52 (3.76-8.11)
Liver or pancreatic	6.87 (4.01-11.78)	6.46 (3.85-10.83)	6.44 (3.85-10.77)	8.57 (5.18-14.18)	7.50 (4.59-12.27)
Other abdominal	2.06 (1.54-2.77)	2.19 (1.66-2.89)	2.28 (1.73-3.01)	2.48 (1.89-3.27)	2.35 (1.79-3.10)
AAA	17.81 (12.14-26.12)	19.34 (14.31-26.14)	18.61 (13.78-25.14)	18.18 (13.49-24.52)	24.36 (18.15-32.68)
Other vascular	7.03 (5.15-9.61)	7.30 (5.48-9.73)	7.57 (5.68-10.09)	8.95 (6.75-11.88)	13.00 (9.84-17.16)
Thoracic	3.46 (2.07-5.79)	3.41 (2.07-5.59)	3.30 (2.01-5.42)	3.38 (2.06-5.54)	2.70 (1.66-4.41)
Retroperitoneal	1.07 (0.53-2.19)	1 [Reference]	1 [Reference]	1 [Reference]	1 [Reference]
eGFR, per 1 mL/min/1.73 m^2^ increase	0.97 (0.97-0.97)	0.97 (0.97-0.97)	0.97 (0.96-0.97)	0.96 (0.96-0.97)	NA
Hemoglobin concentration, per 0.1 g/dL increase	0.99 (0.98-0.99)	0.99 (0.98-0.99)	0.98 (0.98-0.99)	0.98 (0.98-0.99)	NA
Myocardial infarction	1.68 (1.34-2.11)	1.63 (1.32-2.03)	1.63 (1.31-2.02)	NA	NA
Mild liver disease^b^	2.31 (1.64-3.24)	2.32 (1.66-3.24)	2.32 (1.71-3.34)	NA	NA
Moderate or severe liver disease^c^	4.82 (3.47-6.71)	4.96 (3.58-6.85)	4.64 (3.36-6.41)	NA	NA
Albuminuria^d^					
Normal albumin level	1 [Reference]	1 [Reference]	NA	NA	NA
Mild	1.82 (1.47-2.26)	1.88 (1.52-2.33)	NA	NA	NA
Heavy	3.58 (2.85-4.51)	3.74 (2.98-4.69)	NA	NA	NA
Cancer	1.01 (0.80-1.27)	NA	NA	NA	NA
Cerebrovascular disease	0.72 (0.54-0.97)	NA	NA	NA	NA
Congestive heart failure	0.93 (0.73-1.19)	NA	NA	NA	NA
COPD	0.96 (0.79-1.17)	NA	NA	NA	NA
Dementia	0.60 (0.38-0.95)	NA	NA	NA	NA
HIV infection or AIDS	2.00 (0.59-6.83)	NA	NA	NA	NA
Metastatic carcinoma	0.84 (0.59-1.21)	NA	NA	NA	NA
Paraplegia	1.18 (0.64-2.17)	NA	NA	NA	NA
Peptic ulcer disease	1.04 (0.76-1.41)	NA	NA	NA	NA
Peripheral vascular disease	1.14 (0.88-1.49)	NA	NA	NA	NA
Rheumatic or connective tissue disease	1.01 (0.64-1.6)	NA	NA	NA	NA
Diabetes	0.95 (0.78-1.17)	NA	NA	NA	NA
Hypertension	1.04 (0.82-1.33)	NA	NA	NA	NA
Emergent or urgent surgery	1.37 (1.11-1.69)	NA	NA	NA	NA
Intercept, β_0_	0.02 (0.01-0.04)	0.02 (0.01-0.04)	0.05 (0.03-0.08)	0.09 (0.05-0.14)	0.00 (0.00-0.00)

Abbreviations: AAA, abdominal aortic aneurysm; ACR, albumin to creatinine ratio; COPD, chronic obstructive pulmonary disease; eGFR, estimated glomerular filtration rate; NA, not applicable.

SI conversion factor: To convert hemoglobin concentration to grams per liter, multiply by 10.0; and urinary ACR to milligrams per millimole, multiply by 0.113.

^a^ Model 1 (the full model) included 30 variables; model 2, 15 variables selected in at least 80% of samples after bootstrap resampling; model 3, variables from model 2 excluding albuminuria; model 4, age, sex, surgery type, eGFR, and hemoglobin concentration (10 variables); and model 5, age, sex, and surgery type.

^b^ Mild liver disease includes chronic liver diseases and cirrhosis.

^c^ Moderate or severe liver diseases include esophageal varices with or without bleeding, hepatic encephalopathy, portal hypertension, hepatorenal syndrome, and other sequelae of chronic liver disease.

^d^ Normal albumin level was defined by dipstick urinalysis protein negative or an ACR of less than 30 mg/g; mild albuminuria, dipstick urinalysis protein trace or greater than or equal to 1 or ACR from 30 to 300 mg/g; and heavy, dipstick urinalysis protein of greater than or equal to 2 or ACR of more than 300 mg/g.

### Internal Validation

**Figure 2.**
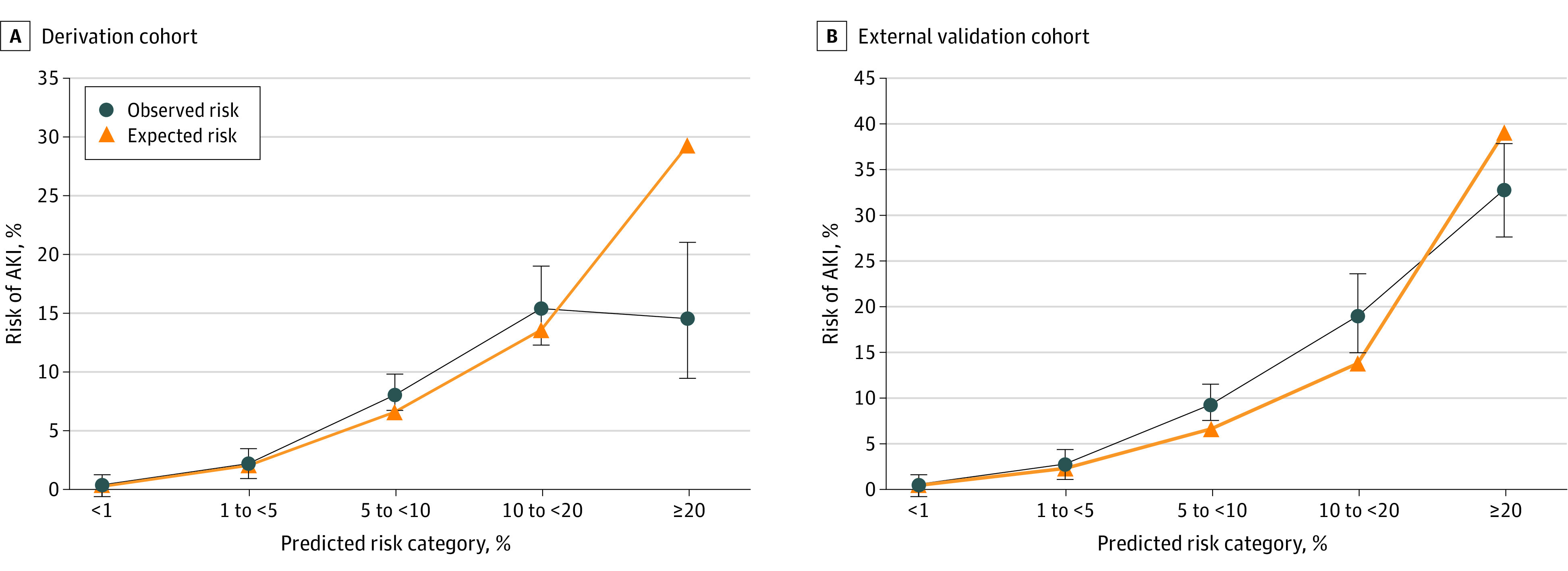
Observed vs Predicted Risk of Acute Kidney Injury Requiring Kidney Replacement Therapy in Model 2 by Clinical Risk Categories in the Derivation and External Validation Cohorts AKI indicates acute kidney injury.

Discrimination, measured by the *C* statistic, ranged from 0.80 (95% CI, 0.78-0.82) for model 5 to 0.89 (95% CI, 0.87-0.90) for model 1 and 0.89 (95% CI, 0.88-0.91) for model 2 (eTable 4 in the Supplement). Model discrimination was similar for models 1 and 2, with the exclusion of most of the comorbidities and elective vs emergent or urgent surgery status; however, the *C* statistic was lower for model 3 (0.87; 95% CI, 0.860.89) and model 4 (0.87; 95% CI, 0.85-0.88), with the removal of albuminuria and additional comorbidities, respectively. The lowest bayesian information criterion was observed for model 2 (5278), indicating the best global model fit after penalization for model complexity. The area under the precision recall curve was similar for models 1 (0.080) and 2 (0.079) and decreased for models 3 (0.069), 4 (0.060), and 5 (0.026). The area under the precision recall curve for the risk index (0.076) was lower than that for model 2. Calibration slope was close to ideal for all models, ranging from 0.98 (95% CI, 0.92-1.06) for model 5 to 0.99 (95% CI, 0.94-1.03) for model 2 in internal validation. Figure 2A shows calibration for model 2 in the derivation cohort for most of the predicted risks, although higher predicted risk vs observed risk occurred at the highest predicted risks (>20%).

Model comparisons based on categorical net reclassification improvement and integrated discrimination improvement are shown in eTable 5 in the Supplement. The inclusion of additional comorbidities in model 1 resulted in few patients with and without the outcome being reclassified into higher and lower risk categories, respectively, compared with model 2, and little improvement observed in the integrated discrimination improvement. Larger changes in net reclassification and discrimination improvement were seen with inclusion of albuminuria in model 2 compared with model 3, from which it was excluded, with an increase of 20 patients (3.8%) with the outcome correctly reclassified into a higher risk category and an increase of 167 patients (0.2%) without the outcome correctly reclassified into a lower risk category. Greater changes in the net reclassification improvement and integrated discrimination improvement were found for model 2 compared with models 4 and 5. Reclassification tables for models 1, 3, 4, and 5 compared with model 2 are shown in eTables 6 to 9 in the Supplement.

An integer-based risk index developed from the best performing model (model 2) (eTable 10 in the Supplement) generated points ranging from 0 to 30, with predicted risks corresponding to each point score ranging from 0.01% (0 points) to 96.7% (30 points) (eTable 11 in the Supplement). The discrimination of the risk index in the derivation cohort was similar to that in model 2, with a *C* statistic of 0.89 (95% CI, 0.87-0.90). The calibration slope for the risk index was 0.97 (95% CI, 0.92-1.03) in the internal validation cohort. Observed and predicted probabilities of AKI requiring KRT for each point value in the risk index are shown in the eFigure in the Supplement.

### External Validation

Model 2 showed excellent discrimination in the external validation cohort, with a *C* statistic of 0.95 (95% CI, 0.95-0.96). On initial application, the calibration slope was 1.28 (95% CI, 1.26-1.30), and the calibration intercept was 1.12 (95% CI, 1.03-1.21). After logistic recalibration of the model coefficients and intercept, the recalibrated model 2 had a calibration slope of 1.00 (95% CI, 0.98-1.02) and calibration intercept of 0.00 (95% CI, –0.07 to 0.07). The area under the precision recall curve for recalibrated model 2 was 0.233, and the recalibrated risk index was 0.218. Figure 2B shows calibration for model 2 in the external validation cohort over a range of predicted risk categories. Regression coefficients for models 2 and 3 and for recalibrated models 2 and 3 are shown in eTable 12 in the Supplement.

The risk index also showed excellent discrimination in the external validation cohort (*C* statistic, 0.95; 95% CI, 0.95-0.96). The original risk index had a calibration slope of 1.22 (95% CI, 1.20-1.24), and calibration intercept of 0.80 (95% CI, 0.72-0.88) for the external validation cohort. With recalibration, the calibration slope improved to 0.96 (95% CI, 0.95-0.98), and the calibration intercept improved to –0.23 (95% CI, –0.30 to –0.16). Observed and predicted probabilities of AKI requiring KRT for each point value of the risk index in the external validation cohort are shown in the eFigure in the Supplement.

A sensitivity of 21.2%, specificity of 99.9%, positive predictive value of 38.1%, and negative predictive value of 99.7% were observed at a predicted risk threshold of 10% or greater (Table 3). An electronic risk calculator for the original and recalibrated models 2 and 3 is available in the eAppendix in the Supplement.

**Table 3.** Predictive Values for Model 2 and the Risk Index in the Derivation and External Validation Cohorts

Predicted risk threshold	Patients, No. (%)^a^	Sensitivity, %	Specificity, %	PPV, %	NPV, %
**Model 2**					
Derivation cohort, %					
≥1	9805 (10.6)	67.5	89.7	3.6	99.8
≥5	1703 (1.8)	34.4	98.3	10.7	99.6
≥10	631 (0.7)	18.1	99.4	15.2	99.5
≥20	158 (0.2)	4.3	99.8	14.5	99.4
External validation cohort, %					
≥1	5063 (0.7)	47.0	99.5	27.4	99.8
≥5	2210 (0.3)	27.4	99.8	36.7	99.7
≥10	1644 (0.2)	21.2	99.9	38.1	99.7
≥20	1218 (0.2)	15.4	99.9	37.3	99.6
**Risk index**					
Derivation cohort, %					
≥1	10 748 (11.7)	70.5	88.7	3.5	99.8
≥5	1713 (1.9)	33.5	98.3	10.3	99.6
≥10	548 (0.6)	15.3	99.5	14.8	99.5
≥20	128 (0.1)	3.4	99.9	14.1	99.4
External validation cohort, %					
≥1	5432 (0.8)	46.8	99.4	25.5	99.8
≥5	2123 (0.3)	24.6	99.8	34.3	99.7
≥10	1323 (0.2)	17.6	99.9	39.2	99.7
≥20	1323 (0.2)	17.6	99.9	39.2	99.7

Abbreviations: NPV, negative predictive value; PPV, positive predictive value.

^a^ Number (percentage) of patients from the cohort with predicted risk of the outcome equal to or greater than the threshold value at each predicted risk threshold.

## Discussion

We developed and internally validated risk prediction models for predicting AKI requiring KRT after major, noncardiac surgery in a large, population-based cohort. We externally validated the best performing model and corresponding risk index in an independent, geographically distinct, population-based cohort, showing excellent discrimination and accuracy of predictions after recalibration. Our models use patient demographics, laboratory data, and comorbidities, which are all readily available variables in preoperative care. This risk index can be implemented before surgery to identify patients at increased risk for AKI requiring KRT after noncardiac surgery. Preoperative risk stratification for severe AKI could be valuable for patient education, potential modification of treatment decisions, and resource allocation because AKI requiring KRT is a serious adverse outcome that is important to patients and is resource intensive for the health care system.

Our final model and risk index showed excellent discrimination and calibration in internal validation.^30,31^ Precision (positive predictive value) and recall (sensitivity) were relatively low, whereas negative predictive values and specificity of the final model were high, as expected, given the low incidence of the outcome. Reduced models excluding albuminuria resulted in some loss of discrimination and poorer stratification into low-, moderate-, and high-risk groups, supporting the prognostic importance of albuminuria,^39,40,41^ a relatively simple laboratory measure that can be routinely incorporated into assessment of risk for AKI. A model without albuminuria performed reasonably well and could be used when albuminuria measurements are not available. The greatest decrease in model performance was observed with a model based on demographic variables and surgery type alone, which highlights the value of multivariable approaches incorporating laboratory measures of eGFR and hemoglobin concentration for AKI risk prediction. External validation of our final model and corresponding risk index showed strong discrimination and excellent calibration after the model was recalibrated to reflect differences in baseline risk and associations between predictors and outcome in the external validation cohort. These findings highlight the importance of recalibrating risk models for application in new populations, which may be particularly important for the outcome of AKI requiring KRT because thresholds for initiating KRT may vary among regions and over time.^42,43^

The variables included in our final model and risk index have been previously associated with AKI, including lower eGFR, greater albuminuria, lower hemoglobin concentration, history of vascular surgery, and presence of heart and liver disease.^27,40,44,45^ We found that increasing age was associated with a lower risk of AKI requiring KRT, which differed from findings of other studies that examined the association with AKI that were not limited to patients who received KRT.^11,25,46^ A reason for these results may be that older patients are less likely to be receive KRT than younger patients or older patients at risk for AKI may be preselected for nonsurgical treatment options.

The development of these models is valuable because little research has been conducted on predicting AKI after noncardiac surgery.^11^ Most published models have been limited to smaller, single-center cohorts of patients undergoing liver transplantation or resection only. Kheterpal et al^27^ developed a risk index for predicting AKI after general surgery using a large national clinical research data set. However, their study predicted the risk of AKI defined as a postoperative increase in creatinine concentration of more than 2 mg/dL (177 μmol/L) or the need for dialysis within 30 days of surgery.^15^ More recently, Park et al^12^ developed and validated the Simple Postoperative AKI Risk (SPARK) index, which can be used for predicting any postoperative AKI or critical AKI, defined as Kidney Disease: Improving Global Outcomes stage 2 or 3 AKI, post-AKI death, or dialysis within 90 days of noncardiac surgery.^12^ Both models differ from our model because they include milder stages of AKI in the outcome as opposed to our model. Our model was developed to predict AKI requiring KRT, which has a greater effect on patient health and health resource utilization.

This study may have implications for patients, care providers, and decision makers. Obtaining individualized information on the risk of AKI requiring KRT may help inform decisions about pursuing a course of treatment or help better prepare patients for an adverse postoperative event. Care providers could use this tool to identify patients at high risk of AKI, which would assist with patient counseling, targeting use of preventative measures to reduce the risk of AKI, and prompting more careful postoperative monitoring of kidney function in patients at high risk. Being able to identify patients at high risk for requiring KRT before surgery may allow for better planning of resource allocation, including a need for preoperative consultation with nephrologists or planning surgical procedures in centers with access to KRT. Additional research is needed to test the clinical impact of the risk prediction tool for perioperative decision-making, including studies to characterize risk thresholds for surgical procedures for which patient preferences or clinicians’ recommendations to undergo or avoid surgery might change, or to identify clinically motivated risk thresholds at which to test interventions for AKI prevention in particular patient populations.

### Limitations

This study has limitations. Although comorbidities were identified from administrative data using validated approaches, these data were not specifically collected for AKI risk prediction, which may have limited the candidate variables available for study; important risk factors for AKI, including disease severity, exposure to periprocedure nephrotoxins, and details on surgical approaches, may not have been included. A proportion of the cohort did not have a preoperative albuminuria measurement, which may have influenced the performance of the models. However, we used an imputation method to address the missing data and found improvement in model performance with inclusion of albuminuria, which provided reassuring findings about the validity of imputing albuminuria in the models. Kidney replacement therapy for patients with AKI may differ based on patient preferences and institutional practices. Thus, the risk models may not be generalizable for use in all locations and patient populations.^47^ The model and risk index may have overestimated absolute risk at high levels of predicted risk (ie, risk index ≥20 points; predicted risk >20%). However, this would be unlikely to influence decisions based on the model if a risk threshold of 10% were used to stratify patients into moderate- vs high-risk status in clinical practice. For example, with a risk threshold of 10% or greater selected to stratify patients at high risk for AKI requiring KRT, the model had a sensitivity of 21.2%, specificity of 99.9%, positive predictive value of 38.1%, and negative predictive value of 99.7%, suggesting that the model may be clinically useful for identifying patients at high risk.

## Conclusions

In this prognostic study, we developed and externally validated a logistic regression model and a risk index for predicting AKI requiring KRT after major, noncardiac surgical procedures that showed excellent performance. This prognostic tool uses readily available preoperative data that may facilitate its implementation for use and assessment in clinical perioperative risk stratification.

## Notes

Supplement. **eTable 1.** Diagnosis and Procedure Codes List for AKI Requiring Kidney Replacement Therapy and Charlson Comorbidities **eTable 2.** ICD-10 CA/CCI Procedure Codes for Surgical Procedures Types **eTable 3.** Baseline Characteristics of the Derivation Cohort by Outcome Status **eTable 4.** Model Performance Statistics in the Derivation / Internal Validation Cohort **eTable 5.** Net Reclassification Improvement and Integrated Discrimination Improvement **eTable 6.** Event and Non-event Reclassification for Model 2 versus Model 1 in the Derivation Cohort **eTable 7.** Event and Non-event Reclassification for Model 3 versus Model 2 in the Derivation Cohort **eTable 8.** Event and Non-event Reclassification for Model 4 versus Model 2 in the Derivation Cohort **eTable 9.** Event and Non-event Reclassification for Model 5 vs Model 2 in the Derivation Cohort **eTable 10.** Development of Risk Index from Model 2 **eTable 11.** Risk Estimates Associated with Point Totals from Risk Index **eTable 12.** Odds Ratios with 95% confidence intervals for Predictive Variables Included in Models 2 and 3 for Alberta and Recalibrated for Ontario **eFigure**. Observed versus Predicted Probability of AKI Requiring Kidney Replacement Therapy Within 14 days of Non-Cardiac Surgery by Risk Index in the Development and External Validation Cohorts **eAppendix.** Risk Calculator for Acute Kidney Injury Requiring Kidney Replacement Therapy After Non-Cardiac Surgery
