# Supplementary material for: Derivation and External Validation of a Risk Index for Predicting Acute Kidney Injury Requiring Kidney Replacement Therapy After Noncardiac Surgery
Source: JAMA Netw Open. 2021 Aug 23;4(8):e2121901. doi: 10.1001/jamanetworkopen.2021.21901 (PMC8383136; doi:10.1001/jamanetworkopen.2021.21901)
Supplement: Supplement. — eTable 1. Diagnosis and Procedure Codes List for AKI Requiring Kidney Replacement Therapy and Charlson Comorbidities eTable 2. ICD-10 CA/CCI Procedure Codes for Surgical Procedures Types eTable 3. Baseline Characteristics of the Derivation Cohort by Outcome Status eTable 4. Model Performance Statistics in the Derivation / Internal Validation Cohort eTable 5. Net Reclassification Improvement and Integrated Discrimination Improvement eTable 6. Event and Non-event Reclassification for Model 2 versus Model 1 in the Derivation Cohort eTable 7. Event and Non-event Reclassification for Model 3 versus Model 2 in the Derivation Cohort eTable 8. Event and Non-event Reclassification for Model 4 versus Model 2 in the Derivation Cohort eTable 9. Event and Non-event Reclassification for Model 5 vs Model 2 in the Derivation Cohort eTable 10. Development of Risk Index from Model 2 eTable 11. Risk Estimates Associated with Point Totals from Risk Index eTable 12. Odds Ratios with 95% confidence intervals for Predictive Variables Included in Models 2 and 3 for Alberta and Recalibrated for Ontario eFigure. Observed versus Predicted Probability of AKI Requiring Kidney Replacement Therapy Within 14 days of Non-Cardiac Surgery by Risk Index in the Development and External Validation Cohorts eAppendix. Risk Calculator for Acute Kidney Injury Requiring Kidney Replacement Therapy After Non-Cardiac Surgery [file jamanetwopen-e2121901-s001.pdf]

Supplementary Online Content

Wilson TA, de Koning L, Quinn RR, et al. Derivation and external validation of a risk index for predicting acute kidney injury requiring kidney replacement therapy after noncardiac surgery. *JAMA Netw Open*. 2021;4(8):e2121901. doi:10.1001/jamanetworkopen.2021.21901

eTable 1. Diagnosis and Procedure Codes List for AKI Requiring Kidney Replacement Therapy and Charlson Comorbidities
eTable 2. ICD-10 CA/CCI Procedure Codes for Surgical Procedures Types
eTable 3. Baseline Characteristics of the Derivation Cohort by Outcome Status
eTable 4. Model Performance Statistics in the Derivation / Internal Validation Cohort
eTable 5. Net Reclassification Improvement and Integrated Discrimination Improvement
eTable 6. Event and Non-event Reclassification for Model 2 versus Model 1 in the Derivation Cohort
eTable 7. Event and Non-event Reclassification for Model 3 versus Model 2 in the Derivation Cohort
eTable 8. Event and Non-event Reclassification for Model 4 versus Model 2 in the Derivation Cohort
eTable 9. Event and Non-event Reclassification for Model 5 versus Model 2 in the Derivation Cohort
eTable 10. Development of Risk Index from Model 2
eTable 11. Risk Estimates Associated with Point Totals from Risk Index
eTable 12. Odds Ratios with 95% confidence intervals for Predictive Variables Included in Models 2 and 3 for Alberta and Recalibrated for Ontario
eFigure. Observed versus Predicted Probability of AKI Requiring Kidney Replacement Therapy Within 14 days of Non-Cardiac Surgery by Risk Index in the Development and External Validation Cohorts
eAppendix. Risk Calculator for Acute Kidney Injury Requiring Kidney Replacement Therapy After Non-Cardiac Surgery

This supplementary material has been provided by the authors to give readers additional information about their work.

© 2021 Wilson TA et al. *JAMA Network Open*.

eTable 1. Diagnosis and Procedure Codes list for AKI Requiring Kidney Replacement Therapy and Charlson Comorbidities

Variable	ICD-9 CM[a]	CCP	ICD-10 CA[a]	CCI
AKI requiring Kidney Replacement Therapy (Alberta)	ICD-9 diagnosis codes 584, 584.5, 584.6, 584.7, 584.8, 584.9 plus one of CCP codes 39.95, V45.1, V56.0, V56.1		ICD-10 diagnosis codes N17, N17.0, N17.1, N17.2, 17.8, N17.9 plus one of CCI codes 1.PZ.21.HQBR, 1.PZ.21.HPD4, 1.PZ.21.HQBS, 1.JQ.53.^^, 1.JT.53.^^	
		13.99A, 13.99B, 13.99AB, 13.99OA	Z99.2, Z49.1, Z49.0 without need for CCI codes	
AKI requiring Kidney Replacement Therapy (Ontario)	OHIP codes for Hemodialysis: R849, G323, G325, G866, 1PZ21HQBR, 51.95, Peritoneal dialysis: G330, G331, 66.98,1PZ21HPD4, or continuous renal replacement therapy: G082, G083, G085, G090, G091, G092, G093, G095, G294, G295, 1PZ21HQBS			
Comorbidities				
Myocardial Infarction	410.x, 412.x		I21.x, I22.x, I25.2	
Mild liver disease	571.2, 571.4–571.6		10 B18.x, K70.0–K70.3, K70.9, K71.3–K71.5, K71.7, K73.x, K74.x, K76.0, K76.2–K76.4, K76.8, K76.9, Z94.4	
Moderate/Severe liver disease	456.0–456.21, 572.2–572.8		I85.0, I85.9, I86.4, I98.2, K70.4, K71.1, K72.1, K72.9, K76.5, K76.6, K76.7	
Hypertension	401, 402, 403, 404, 405		I.10, I.11, I12, I.13, I.14, I.15	
Peripheral Vascular Disease	440.2		I70.2	
Congestive Heart Failure	398.91, 402.01, 402.11, 402.91, 404.01, 404.03, 404.11, 404.13, 404.91, 404.93, 425.4–425.9, 428.x		I09.9, I11.0, I13.0, I13.2, I25.5, I42.0, I42.5–I42.9, I43.x, I50.x, P29.0	
Peptic Ulcer Disease	531.7, 531.9, 532.7, 532.9, 533.7, 533.9, 534.7, 534.9		K25.7, K25.9, K26.7, K26.9, K27.7, K27.9, K28.7, K28.9	
Diabetes mellitus	250.x		E10 – E14	
Chronic Obstructive Pulmonary Disease	490.x–505.x, 506.4 I27.8,		10 I27.9, J40.x–J47.x, J60.x–J67.x, J68.4, J70.1, J70.3	

© 2021 Wilson TA et al. *JAMA Network Open*.

eTable 1. Diagnosis and Procedure Codes list for AKI Requiring Kidney Replacement Therapy and Charlson Comorbidities (continued)

Cerebrovascular Disease	430.x–438.x		G45.x, G46.x, H34.0, I60.x–I69.x	
Cancer	140.x–172.x, 174.x.–195.8, 200.x–208.x		10C00.x–C26.x, C30.x–C34.x, C37.x–C41.x, C43.x, C45.x–C58.x, C60.x–C76.x, C81.x–C85.x, C88.x, C90.x–C97.x	
Metastatic Carcinoma	196.x – 199.1		C77.x – C80.x	
Rheumatic / Connective Tissue	710.0, 710.1, 710.4,714.0–714.2, 714.81, 725.x		M05.x, M06.x, M31.5, M32.x–M34.x, M35.1, M35.3, M36.0	
Paraplegia or Hemiplegia	344.1, 342.x		G04.1, G11.4, G80.1, G80.2, G81.x, G82.x, G83.0–G83.4, G83.9	
HIV / AIDS	042.x–044.x		B20.x–B22.x, B24.x	
Dementia	290.x		F00.x–F03.x, F05.1, G30.x, G31.1	
Hypertension	401-405		I.10, I.11, I12, I.13, I.14, I.15	

[a] ICD-9 CM codes were used for physician claims data, ICD-10 CM / CCI codes were used for hospital discharge abstract data
Abbreviations: ICD - International Classification of Diseases, CCP – Canadian Classification of Procedures, CCI - Canadian Classification of Health Interventions (CCI), AKI – Acute kidney injury

© 2021 Wilson TA et al. *JAMA Network Open*.

eTable 2. ICD-10 CA/CCI Procedure Codes for Surgical Procedures Types

Surgical Procedure Types	CCI Codes
Musculoskeletal	1VZ94LA; 1VZ70LA; 1VS80; 1VS72; 1VS58; 1VR80; 1VR72; 1VR58; 1VR57; 1VQ93; 1VQ91; 1VQ87; 1VQ83; 1VQ82; 1VQ80; 1VQ79; 1VQ74; 1VQ73LA; 1VQ58; 1VP89; 1VP87; 1VP80; 1VP74; 1VP73LA; 1VP72; 1VP53; 1VN; 1VM; 1VL; 1VK89; 1VK87; 1VK80; 1VG93; 1VG87; 1VG83; 1VG80; 1VG75; 1VG74; 1VG73LA; 1VG72LA; 1VG72DA; 1VG58DA; 1VG57; 1VG53; 1VE80; 1VE72; 1VE58; 1VD87; 1VD80; 1VD72; 1VD58; 1VD57; 1VC93; 1VC91; 1VC87; 1VC83; 1VC82; 1VC80; 1VC79; 1VC74; 1VC73LA; 1VC58; 1VA93; 1VA87; 1VA83; 1VA80; 1VA75; 1VA74; 1VA73LA; 1VA72LA; 1VA72DA; 1VA58DA; 1VA57; 1VA53; 1UV80; 1UV72; 1UV58; 1UU84; 1UU80; 1UU72; 1UU53; 1UT84; 1UT80; 1UT72LA; 1UT53; 1US80; 1US72; 1US58; 1UK93; 1UK87; 1UK80; 1UK75; 1UK74; 1UK73LA; 1UK72LA; 1UK53; 1UJ93; 1UJ87; 1UJ82; 1UJ80; 1UJ75; 1UJ74; 1UJ73LA; 1UJ71; 1UJ58; 1UG93; 1UG87; 1UG80; 1UG75; 1UG74; 1UG73LA; 1UG72LA; 1UG72JAHB; 1UG72JAAZ; 1UG57; 1UG53; 1UF93; 1UF87; 1UF84; 1UF82; 1UF80; 1UF79; 1UF74; 1UF73LA; 1UC89; 1UC87; 1UC82; 1UC80; 1UC79; 1C75; 1UC74; 1UC73LA; 1UC72; 1UC57; 1UC53; 1UB93; 1UB87; 1UB83; 1UB80; 1UB75; 1UB74; 1UB73LA; 1UB72LA; 1UB72DA; 1UB58; 1UB57; 1UB53; 1TZ94LA; 1TZ70LA; 1TV93; 1TV91; 1TV87; 1TV84; 1TV83; 1TV82; 1TV80; 1TV79; 1TV74; 1TV73LA; 1TV58; 1TS80; 1TS72; 1TS58; 1TQ80; 1TQ72; 1TQ58; 1TQ57; 1TM93; 1TM87; 1TM83; 1TM80; 1TM75; 1TM74; 1TM73LA; 1TM72LA; 1TM72DA; 1TM58; 1TM57; 1TM53; 1TK93; 1TK91; 1TK87; 1TK83; 1TK82; 1TK80; 1TK79; 1TK74; 1TK73LA; 1TK58; 1TH; 1TF80; 1TF72; 1TF58; 1TF57; 1TC80; 1TC72; 1TC57; 1TB87; 1TB80; 1TB74; 1TB72LA; 1TB72JAHB; 1TB72JAAZ; 1TB72DA; 1TA93; 1TA87; 1TA83; 1TA80; 1TA75; 1TA74; 1TA73LA; 1TA72LA; 1TA72DA; 1TA58; 1TA57; 1TA53; 1SY87; 1SY84; 1SY80; 1SY72; 1SY58; 1SY57; 1SY53; 1SW87; 1SW74; 1SQ93; 1SQ91; 1SQ87; 1SQ83; 1SQ80; 1SQ74; 1SQ58; 1SQ53; 1SN93; 1SN87; 1SN75; 1SN74; 1SN72; 1SN58; 1SM87; 1SM80; 1SM74; 1SM73LA; 1SL91; 1SL89; 1SL87; 1SL80; 1SL74; 1SL73LA; 1SL58; 1SK87; 1SK80; 1SK74; 1SK73LA; 1SG87; 1SG80; 1SG72WK; 1SG72WJ; 1SG58; 1SF89; 1SF87; 1SF80; 1SF75; 1SF74; 1SF73PF; 1SE89; 1SE87PF; 1SE59LAGX; 1SE53; 1SC89; 1SC87; 1SC80; 1SC75; 1SC74; 1SC72JAHB; 1SC72JAAZ; 1SA89; 1SA80; 1SA75; 1SA74
Colorectal	1NM56LA; 1NM58; 1NM74; 1NM76RE; 1NM76RN; 1NM77RS; 1NM80LA; 1NM82RN: 1NM82RS; 1NM87LA; 1NM89RN; 1NM89TF; 1NM91R; 1NM91T; CCP: 57.04, 57.2, 57.33, 57.5, 57.6, 57.8, 58.03, 58.04, 58.1, 58.41, 58.43, 58.44, 58.51, 58.53, 58.61, 58.64, 58.65, 58.75, 58.76, 58.79, 58.81, 58.83, 58.91, 58.92, 58.94, 58.96, 60.0, 60.1, 603, 60.4, 60.5, 60.6
Liver / Pancreatic	1OA58; 1OA59LAGX; 1OA74; 1OA87LA; 1OJ56; 1OJ76VK; 1OJ76VL; 1OJ83; 1OJ85; 1OJ87; 1OJ89; 1OK58; 1OK85; 1OK87; 1OK89; 1OK91; CCP: 62.0, 62.1, 62.2, 62.3, 62.5, 64

© 2021 Wilson TA et al. *JAMA Network Open*.

eTable 2. ICD-10 CA/CCI Procedure Codes for Surgical Procedures Types (continued)

Other Abdominal	1MG87; 1MG89; 1MJ87; 1MJ89; 1MJ91; 1MP50; 1MP51; 1MP59; 1MP76; 1MP80; 1MP87; 1NF80DAXXE; 1NF80DAXXN; 1NF80LA; 1NF80LAXXE; 1NF80LAXXN; 1NF82; 1NF84; 1NF86; 1NF87; 1NF89; 1NF90; 1NF91; 1NF92; 1NK53DATS; 1NK53LAQB; 1NK53LATS; 1NK56DA; 1NK56LA; 1NK58; 1NK74; 1NK76; 1NK77; 1NK80; 1NK82; 1NK84; 1NK85; 1NK87DA; 1NK87DN; 1NK87DP; 1NK87DX; 1NK87DY; 1NK87LA; 1NK87RE; 1NK87RF; 1NK87TF; 1NK87TG; 1NM56DA; 1NM76DF; 1NM76DN; 1NM77EP; 1NM80DA; 1NM82EP; 1NM87 (except 1NM87LA); 1NM89D; 1NM91D; 1NP58; 1NP72; 1NP73LA; 1NP85; 1NP86; 1NV89; 1OA53; 1OA59DAGX; 1OA85; 1OA87DA; 1OB59DAGX; 1OB59LAGX; 1OB74; 1OB83; 1OB85; 1OB87; 1OB89; 1OD57; 1OD76; 1OD80; 1OD86; 1OD89; 1OE57DAAG; 1OE57DAAM; 1OE57DAAS; 1OE57DAAZ; 1OE57DABD; 1OE57DAGX; 1OE57HAAG; 1OE57HAAM; 1OE57HAAS; 1OE57HAAZ; 1OE57HABD; 1OE57HAGX; 1OE57LAAG; 1OE57LAAM; 1OE57LAAS; 1OE57LAAZ; 1OE57LABD; 1OE57LAGX; 1OE59KQ; 1OE76; 1OE80; 1OE84; 1OE86; 1OE87; 1OE89; 1OJ53; 1OJ76BR; 1OJ76EG; 1OJ76EH; 1OT53; 1OT56; 1OT58; 1OT70; 1OT72; 1OT80; 1OT87; 1OT91; 1OW12; 1OW80DA; 1OW80LA; 1OW87; 1OW89; 1OZ94LA
Abdominal Aortic Aneurysm	ICD-10 code I71.4 plus one of CCI codes 1KA80LA-XXN or 1KA80GQ-NRN or CCI: 1KA76MZ-XXN or 1KA76NB-XXN or 1KA50GQ-OA (GQ-BD/GS-BD) or 1KE50GQ-OA (GQ-BD/GS-BD) without need for ICD-10 diagnostic code
Other Vascular	1ID57; 1ID76; 1ID80; 1ID82; 1ID86; 1ID87; 1JD53; 1JD59; 1JD89; 1JE50; 1JE51; 1JE57; 1JE58; 1JE59; 1JE76; 1JE80; 1JE87; 1JJ50; 1JJ51; 1JJ57; 1JJ58; 1JJ76; 1JJ80; 1JJ83; 1JJ87; 1JK50; 1JK51; 1JK57; 1JK58; 1JK76; 1JK80; 1JK87; 1JL50; 1JL51; 1JL57; 1JL58; 1JL80; 1JL87; 1JM50; 1JM51; 1JM57; 1JM58; 1JM76; 1JM80; 1JM82; 1JM87; 1JQ50; 1JQ51; 1JQ57; 1JQ80; 1JQ87; 1JT50; 1JT51; 1JT57; 1JT58; 1JT80; 1JT87; 1JU; 1JY50; 1JY51; 1JY57; 1JY76; 1JY80; 1JY87; 1KA50; 1KA53; 1KA57; 1KA58; 1KA76; 1KA80; 1KA82; 1KA87; 1KE50; 1KE51; 1KE57; 1KE58; 1KE76; 1KE80; 1KE87; 1KG50; 1KG51; 1KG57; 1KG58; 1KG76; 1KG80; 1KG82; 1KG87; 1KQ; 1KR34; 1KR50; 1KR51; 1KR53; 1KR57; 1KR58; 1KR59; 1KR76; 1KR78; 1KR80; 1KR83; 1KR87; 1KT50; 1KT51; 1KT58; 1KT76; 1KT80; 1KT82; 1KT87; 1KV53; 1KV80; 1KX53; 1KX80; 1KY; 1KY76LA; 1KY76LASJ; 1KY76LAXXA; 1KY76LAXXL; 1KY76LAXXN 1KZ
Thoracic	1GM50LA; 1GM56LA; 1GM80DAXXE; 1GM80LA; 1GM80LAXXE; 1GM80LAXXG; 1GM86; 1GM87; 1GN; 1GR; 1GT56; 1GT58; 1GT59; 1GT78; 1GT80; 1GT85; 1GT87; 1GT89; 1GT91; 1GV56; 1GV59DAGX; 1GV59DAZ9; 1GV59LAGX; 1GV76; 1GV80; 1GV87; 1GV89; 1GW56; 1GW59; 1GW87; 1GX78; 1GX80; 1GX86; 1GX87; 1GY56; 1GY70; 1GY72; 1GY86; 1GY94DA; 1GY94LA; 1ME87; 1ME89; 1MF87; 1MM; 1MN50; 1MN51; 1MN59; 1MN74; 1MN76; 1MN77; 1MN80; 1MN87; 1NA56DB; 1NA56DBXXF; 1NA56DBXXG; 1NA56EZ; 1NA56EZXXG; 1NA56FA; 1NA56FAXXF; 1NA56FAXXG; 1NA56LB; 1NA56LBXXF; 1NA56LBXXG; 1NA56LP; 1NA56LPXXF; 1NA56LPXXG; 1NA56QB; 1NA56QBXXF; 1NA56QBXXG; 1NA56QFXXF; 1NA56QFXXG; 1NA72; 1NA74; 1NA76; 1NA77; 1NA80; 1NA82; 1NA84; 1NA86; 1NA87; 1NA88; 1NA89; 1NA90; 1NA91; 1NA92
Retroperitoneal	1PB87; 1PB89; 1PC51LALV; 1PC56; 1PC59DAGX; 1PC59LAGX; 1PC80; 1PC82; 1PC83; 1PC85; 1PC86; 1PE50DABD; 1PE50DABF; 1PE50DABJ; 1PE56DA; 1PE56LA; 1PE59LAAG; 1PE59LAGX; 1PE76; 1PE77; 1PE80; 1PE82; 1PE87; 1PE89; 1PG50DABD; 1PG50DABF; 1PG50DABJ; 1PG50LABJ; 1PG52DA; 1PG56DA; 1PG56LA; 1PG57DAGX; 1PG57LAAM; 1PG57LAGX; 1PG59DAAG; 1PG59DAAS; 1PG59DAAT; 1PG59DAAZ; 1PG59DAGX; 1PG59KQAP;

© 2021 Wilson TA et al. *JAMA Network Open*.

	1PG59KQAQ; 1PG59KQAR; 1PG59LAAG; 1PG59LAGX; 1PG72; 1PG74; 1PG76; 1PG77; 1PG80DA; 1PG80LA; 1PG80LAXXE; 1PG80LD; 1PG82; 1PG86; 1PG87; 1PG98; 1PL50LAGX; 1PL53; 1PL59LAAD; 1PL59LAAG; 1PL59LAAS; 1PL59LAAZ; 1PL59LAGX; 1PL72LA; 1PL72LAAG; 1PL74; 1PL80; 1PL87; 1PM56DA; 1PM56LA; 1PM57DAGX; 1PM57LAGX; 1PM58LA; 1PM59DAAG; 1PM59DAAS; 1PM59DAAT; 1PM59DAAZ; 1PM59DAGX; 1PM59DAX7; 1PM59KQAP; 1PM59KQAQ; 1PM59KQAR; 1PM72; 1PM77; 1PM79; 1PM80AF; 1PM80FJ; 1PM80LA; 1PM82; 1PM84; 1PM86; 1PM87LA; 1PM89LA; 1PM90; 1PM91; 1PM92

© 2021 Wilson TA et al. *JAMA Network Open*.

eTable 3. Baseline Characteristics of the Derivation Cohort by Outcome Status

Variable	Total Cohort (n=92,114)	With AKI requiring KRT (n=529)	Without AKI requiring KRT (n=91,585)
Age, mean (SD), (years)	62.3 (18.0)	63.0 (15.2)	62.3 (18.0)
Sex (Male)	44,034 (47.8%)	347 (65.6%)	43,687 (47.7%)
Emergent / Urgent Surgery	50,231 (54.5%)	352 (66.5%)	49,879 (54.5%)
**Surgery Type**			
Musculoskeletal	42,864	76 (14.4%)	42,788 (46.7%)
Colorectal	3,585	38 (7.2%)	3,547 (3.9%)
Liver & Pancreas	1,371	20 (3.8%)	1,351 (1.5%)
Other Abdominal	30,346	132 (25.0%)	30,214 (33.0%)
AAA	2,217	108 (20.4%)	2,109 (2.3%)
Other Vascular	5,036	126 (23.8%)	4,910 (5.4%)
Thoracic	3,783	20 (3.8%)	3,763 (4.1%)
Retroperitoneal	2,912	9 (1.7%)	2,903 (3.2%)
**Comorbidities**			
Cancer	25,397	149 (28.2%%)	25,248 (27.6%)
Cerebrovascular Disease	8,957	62 (11.7%)	8,895 (9.7%)
Congestive Heart Failure	10,077	112 (21.2%)%)	9,965 (10.9%)
COPD	24,027	179 (33.8%)	23,848 (26.0%)
Dementia	6,443	21 (4.0%)	6,422 (7.0%)
HIV / AIDS	163	3 (0.6%)	160 (0.2%)
Metastatic Carcinoma	8,489	41 (7.8%)	8,448 (9.2%)
Mild Liver Disease	3,033	43 (8.1%)	2,990 (3.3%)
Moderate / Severe Liver Disease	1,095	56 (10.6%)	1,039 (1.1%)
Myocardial Infarction	8,354	130 (24.6%)	8,224 (9.0%)
Paraplegia or Hemiplegia	1,724	12 (2.3%)	1,712 (1.9%)
Peptic Ulcer Disease	5,331	51 (9.6%)	5,280 (5.8%)
Peripheral Vascular Disease	10,587	206 (38.9%)	10,381 (11.3%)
Rheumatic / Connective Tissue	3,702	21 (4.0%)	3,681 (4.0%)
Diabetes mellitus	18,343	158 (29.9%)	18,185 (19.9%)
Hypertension	11,488	87 (16.5%)	11,401 (12.5%)
**Laboratory Measures**			
eGFR, mean (SD), (mL/min/1.73m^2)	79.3 (24.8)	57.9 (29.8)	79.4 (24.7)
Hemoglobin, mean (SD), (g/L)	127.3 (22.1)	112.3 (26.6)	127.4 (22.0)
Albuminuria[a] Normal	49,427	161 (30.4%)	49,266 (53.8%)
Mild	13,545	115 (21.7%)	13,430 (14.7%)
Heavy	4,833	120 (22.7%)	4,713 (5.2%)
Unmeasured	24,309	133 (25.1%)	24,176 (26.4%)
Albuminuria[a] Imputed Normal	67,732	213 (40.3%)	67,519 (73.7%)
Mild	17,946	161 (30.4%)	17,785 (19.4%)
Heavy	6,436	155 (29.3%)	6,281 (6.9%)

[a] Normal albuminuria is defined by dipstick urinalysis protein negative or albumin:creatinine ratio (ACR) of <30mg/g; mild, dipstick urinalysis protein trace or 1+ or ACR from 30 - 300mg/g; and heavy, dipstick urinalysis protein of 2+ or higher or ACR of more than 300mg/g. To convert urine ACR to mg/mmol, multiply by 0.113.

Abbreviations: SD – standard deviation, AAA - Abdominal Aortic Aneurysm, COPD – chronic obstructive pulmonary disease, eGFR – estimated glomerular filtration rate

© 2021 Wilson TA et al. *JAMA Network Open*.

eTable 4. Model Performance Statistics in the Derivation / Internal Validation Cohort

	Model 1	Model 2	Model 3	Model 4	Model 5	Risk Index
Apparent C-statistic	0.89	0.89	0.88	0.87	0.80	0.89
Optimism adjusted C-statistic (95% CI)	0.89 (0.87, 0.90)	0.89 (0.88, 0.91)	0.87 (0.86, 0.89)	0.87 (0.85, 0.88)	0.80 (0.78, 0.82)	0.89 (0.87, 0.90)
Bayesian Information Criterion (BIC)[a]	5,426	5,278	5,379	5,443	5,950	n/a
Calibration slope (95% CI)	0.98 (0.93, 1.03)	0.99 (0.94, 1.03)	0.99 (0.94, 1.04)	0.99 (0.94, 1.04)	0.98 (0.92, 1.06)	0.97 (0.92, 1.02)
Calibration intercept (95% CI)	-0.08 (-0.31, 0.14)	-0.05 (-0.24, 0.14)	-0.04 (-0.24, 0.17)	-0.04 (-0.27, 0.19)	-0.05 (-0.40, 0.29)	-0.09 (-0.31, 0.13)
Area Under Precision Recall Curve[b]	0.080	0.079	0.069	0.060	0.026	0.076

[a] Null value for the BIC was 6,525. Lower values represent better model fit

[b] Baseline values for the area under the precision recall curve was 0.0057. Higher values indicate better precision-recall.

Abbreviation: CI – confidence interval

© 2021 Wilson TA et al. *JAMA Network Open*.

eTable 5. Net Reclassification Improvement and Integrated Discrimination Improvement

Models	NRI, Number of Patients		Overall NRI	IDI
	With AKI requiring KRT (n=529)	Without AKI requiring KRT (n=91,585)		
M1 improvement over M2	16 (3.0%)	36 (<0.1%)	3.0 %	0.2 %
M2 improvement over M3	20 (3.8%)	167 (0.2%)	4.0 %	0.7 %
M2 improvement over M4	64 (12.1%)	609 (0.7%)	12.8 %	1.2 %
M2 improvement over M5	191 (36.1%)	84 (0.1%)	36.2 %	3.5 %

NRI – net reclassification improvement index, IDI – integrated discrimination improvement index
Risk categories for categorical NRI are <1%, 1-<5%, 5-<10%, 10-<20%, ≥20%

© 2021 Wilson TA et al. *JAMA Network Open*.

eTable 6. – Event and Non-event Reclassification for Model 2 versus Model 1 in the Derivation Cohort

**Risk Model 1**	**Risk Model 2**					
	<1%	1-<5%	5-<10%	10-<20%	≥20%	Total
**Events**						
<1%	156	9	0	0	0	165
1-<5%	16	158	7	1	0	182
5-<10%	0	8	69	4	0	81
10-<20%	0	0	10	63	2	75
≥20%	0	0	0	5	21	26
Total	172	175	86	73	23	529
**Non-Events**						
<1%	81,343	845	0	0	0	82,188
1-<5%	794	6,932	145	1	0	7,872
5-<10%	0	150	772	69	0	991
10-<20%	0	0	69	302	17	388
≥20%	0	0	0	28	118	146
Total	82,137	7,927	986	400	135	91,585

© 2021 Wilson TA et al. *JAMA Network Open*.

eTable 7. – Event and Non-event Reclassification for Model 3 versus Model 2 in the Derivation Cohort

Risk Model 3	Risk Model 2					
	<1%	1-<5%	5-<10%	10-<20%	≥20%	Total
**Events**						
<1%	144	23	0	0	0	167
1-<5%	28	134	24	0	0	186
5-<10%	0	18	57	29	6	110
10-<20%	0	0	5	29	4	38
≥20%	0	0	0	15	13	28
Total	172	175	86	73	23	529
**Non-Events**						
<1%	80,447	1,180	4	0	0	81,631
1-<5%	1,690	6,386	333	162	22	8,593
5-<10%	0	361	514	205	45	1,125
10-<20%	0	0	135	33	68	236
≥20%	0	0	0	0	0	0
Total	82,137	7,927	986	400	135	91,585

© 2021 Wilson TA et al. *JAMA Network Open*.

eTable 8. – Event and Non-event Reclassification for Model 4 versus Model 2 in the Derivation Cohort

Risk Model 4	Risk Model 2					
	<1%	1-<5%	5-<10%	10-<20%	≥20%	Total
Events						
<1%	143	41	1	0	0	185
1-<5%	29	115	41	15	4	204
5-<10%	0	17	28	23	3	71
10-<20%	0	2	16	25	10	53
≥20%	0	0	0	10	6	16
Total	172	175	86	73	23	529
Non-Events						
<1%	79,262	2,118	25	0	0	81,405
1-<5%	2,875	5,290	478	102	5	8,750
5-<10%	0	499	328	155	29	1,011
10-<20%	0	20	155	115	56	346
≥20%	0	0	0	28	45	73
Total	82,137	7,927	986	400	135	91,585

© 2021 Wilson TA et al. *JAMA Network Open*.

eTable 9. – Event and Non-event Reclassification for Model 5 versus Model 2 in the Derivation Cohort

Risk Model 5	Risk Model 2					
	<1%	1-<5%	5-<10%	10-<20%	≥20%	Total
**Events**						
<1%	131	77	21	23	0	252
1-<5%	41	83	53	43	21	241
5-<10%	0	15	12	7	2	36
10-<20%	0	0	0	0	0	0
≥20%	0	0	0	0	0	0
Total	172	175	86	73	23	529
**Non-Events**						
<1%	77,381	3,724	243	70	0	81,418
1-<5%	4,740	3,765	608	261	106	9,480
5-<10%	16	438	135	69	29	687
10-<20%	0	0	0	0	0	0
≥20%	0	0	0	0	0	0
Total	82,137	7,927	986	400	135	91,585

© 2021 Wilson TA et al. *JAMA Network Open*.

eTable 10. Development of Risk Index from Model 2

Risk Factor	Categories	Reference Value	Regression Coefficient	Distance from Reference Category in Regression Units	Points
Intercept			-3.8420		
Age less than 40	No	0	0	0	0
	Yes	1	1.3165	1.6851	3
Age 40 to 70	No	0	0	0	0
	Yes	1	0.7277	0.7277	2
Sex[a]	Female	0	0	0	0
	Male	1	0.4364	0.4364	1
Colorectal Surgery	No	0	0	0	0
	Yes	1	1.5802	1.5802	4
Liver / Pancreatic surgery	No	0	0	0	0
	Yes	1	1.8652	1.8652	4
Abdominal surgery	No	0	0	0	0
	Yes	1	0.7826	0.7826	2
AAA surgery	No	0	0	0	0
	Yes	1	2.9622	2.9622	7
Vascular surgery	No	0	0	0	0
	Yes	1	1.9876	1.9876	5
Thoracic surgery	No	0	0	0	0
	Yes	1	1.2253	1.2253	3
eGFR	120 or greater	126	-0.0300 per unit decrease	0	0
	105-119	112.5		0.4049	1
	90-104	97.5		0.8548	2
	75-89	82.5		1.3047	3
	60-74	67.5		1.7546	4
	45-59	52.5		2.2045	5
	30-44	37.5		2.6544	6
	20-29	25		3.0293	7
	10-19	15		3.3292	8
Hemoglobin	140 or greater	165	-0.0127 per unit decrease	0	0
	110-139	125		0.5074	1
	60-109	85		1.0149	2
	30-59	45		1.5223	3
Albuminuria Mild	No	0		0	0
	Yes	1	0.6305	0.6305	1
Albuminuria Heavy	No	0		0	0
	Yes	1	1.3189	1.3189	3
Charlson Myocardial infarction	No	0		0	0
	Yes	1	0.4912	0.4912	1
Charlson Mild liver disease	No	0		0	0
	Yes	1	0.8408	0.8408	2
Charlson Moderate / Severe liver disease	No	0		0	0
	Yes	1	1.6004	1.6004	4

[a] Sex regression coefficient used to standardize all other coefficients

Abbreviations: AAA - Abdominal Aortic Aneurysm, eGFR – estimated glomerular filtration rate

© 2021 Wilson TA et al. *JAMA Network Open*.

eTable 11. Risk Estimates Associated with Point Totals from Risk Index

Point Total	Estimate of Risk Derivation Cohort	Estimate of Risk External Validation Cohort
0	0.006%	0.001%
1	0.009%	0.002%
2	0.014%	0.004%
3	0.022%	0.007%
4	0.034%	0.011%
5	0.053%	0.020%
6	0.083%	0.035%
7	0.128%	0.061%
8	0.197%	0.106%
9	0.305%	0.186%
10	0.471%	0.325%
11	0.727%	0.566%
12	1.120%	0.986%
13	1.722%	1.711%
14	2.639%	2.953%
15	4.025%	5.051%
16	6.093%	8.509%
17	9.123%	13.985%
18	13.444%	22.132%
19	19.374%	33.195%
20	27.101%	46.486%
21	36.515%	60.295%
22	47.086%	72.639%
23	57.925%	82.273%
24	68.051%	89.028%
25	76.719%	93.414%
26	83.602%	96.124%
27	88.748%	97.745%
28	92.426%	98.698%
29	94.970%	99.251%
30	96.690%	99.570%

© 2021 Wilson TA et al. *JAMA Network Open*.

eTable 12. Odds Ratios with 95% confidence intervals for Predictive Variables Included in Models 2 and 3 for Alberta and Recalibrated for Ontario

Predictive Variable	Model 2 (Alberta)	Model 2 Recalibrated (Ontario)	Model 3 (Alberta)	Model 3 (Ontario)
Age < 40 years	3.73 (2.61, 5.33)	5.39 (3.41, 8.52)	4.58 (3.21, 6.53)	7.34 (5.14, 10.46)
40 to < 70 years	2.07 (1.69, 2.53)	2.54 (1.96, 3.28)	2.23 (1.82, 2.72)	2.85 (2.34, 3.49)
≥ 70 years	1 [Reference]	1 [Reference]	1 [Reference]	1 [Reference]
Sex (Male)	1.55 (1.28, 1.87)	1.75 (1.37, 2.23)	1.73 (1.43, 2.09)	2.04 (1.69, 2.47)
Surgery Type Musculoskeletal	1 [Reference]	1 [Reference]	1 [Reference]	1 [Reference]
Colorectal	4.86 (3.28, 7.18)	7.56 (4.57, 12.47)	5.08 (3.44, 7.50)	8.41 (5.7, 12.42)
Liver / Pancreatic	6.46 (3.85, 10.83)	10.89 (5.62, 21.1)	6.44 (3.85, 10.77)	11.47 (6.86, 19.19)
Other abdominal	2.19 (1.66, 2.89)	2.72 (1.91, 3.89)	2.28 (1.73, 3.01)	2.95 (2.24, 3.89)
AAA	19.34 (14.31, 26.14)	44.33 (30.15, 65.19)	18.61 (13.78, 25.14)	46.08 (34.12, 62.23)
Other vascular	7.30 (5.48, 9.73)	12.73 (8.82, 18.40)	7.57 (5.68, 10.09)	14.19 (10.64, 18.91)
Thoracic	3.41 (2.07, 5.59)	4.80 (2.54, 9.05)	3.30 (2.01, 5.42)	4.78 (2.92, 7.84)
Retroperitoneal	1 [Reference]	1 [Reference]	1 [Reference]	1 [Reference]
eGFR (per 1 mL/min/1.73m^2 increase)	0.97 (0.97, 0.97)	0.96 (0.96, 0.96)	0.97 (0.96, 0.97)	0.95 (0.95, 0.96)
Hemoglobin (per 0.1 g/dL increase)	0.99 (0.98, 0.99)	0.98 (0.97, 0.99)	0.98 (0.98, 0.99)	0.98 (0.98, 0.98)
Myocardial infarction	1.63 (1.32, 2.03)	1.88 (1.43, 2.48)	1.63 (1.31, 2.02)	1.89 (1.53, 2.35)
Mild liver disease	2.32 (1.66, 3.24)	2.93 (1.91, 4.5)	2.32 (1.71, 3.34)	3.14 (2.25, 4.38)
Moderate / Severe liver disease	4.96 (3.58, 6.85)	7.76 (5.12, 11.74)	4.96 (3.36, 6.41)	7.47 (5.41, 10.31)
Albuminuria[a] Normal	1 [Reference]	1 [Reference]		
Mild	1.88 (1.52, 2.33)	2.24 (1.71, 2.95)		
Heavy	3.74 (2.98, 4.69)	5.41 (4.05, 7.23)		
Intercept (β_0)	0.02 (0.01, 0.04)	0.01 (0.00, 0.02)	0.05 (0.03, 0.08)	0.07 (0.04, 0.11)

[a] Normal albuminuria is defined by dipstick urinalysis protein negative or albumin:creatinine ratio (ACR) of <30mg/g; mild, dipstick urinalysis protein trace or 1+ or ACR from 30 - 300mg/g; and heavy, dipstick urinalysis protein of 2+ or higher or ACR of more than 300mg/g. To convert urine ACR to mg/mmol, multiply by 0.113.

© 2021 Wilson TA et al. *JAMA Network Open*.

Figure 1A. Observed versus Predicted Probability of AKI requiring Kidney Replacement Therapy within 14 days of Non-Cardiac Surgery by Risk Index in the Development Validation Cohort

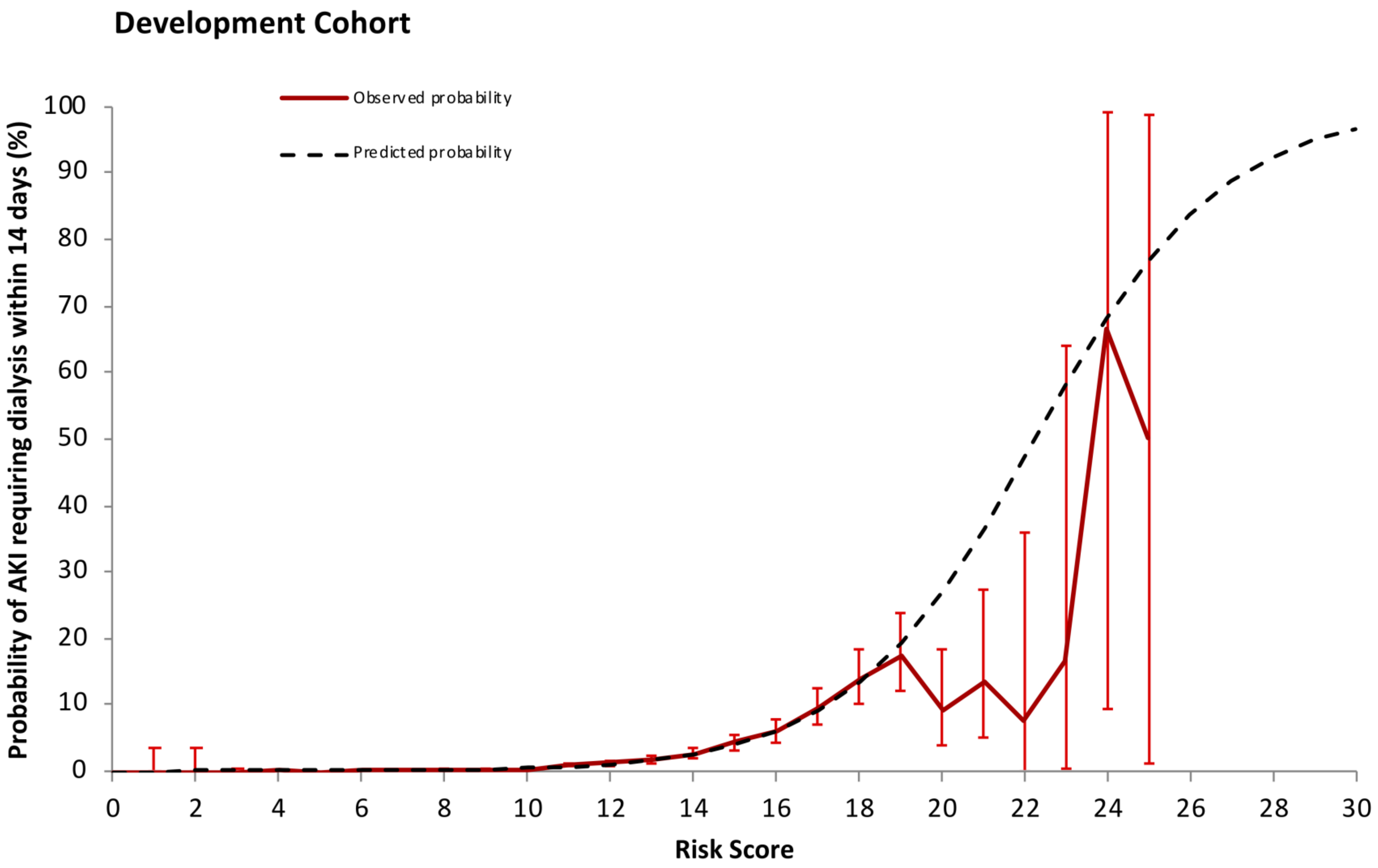

© 2021 Wilson TA et al. *JAMA Network Open*.

Figure 1B. Observed versus Predicted Probability of AKI requiring Kidney Replacement Therapy within 14 days of Non-Cardiac Surgery by Risk Index in the External Validation Cohort

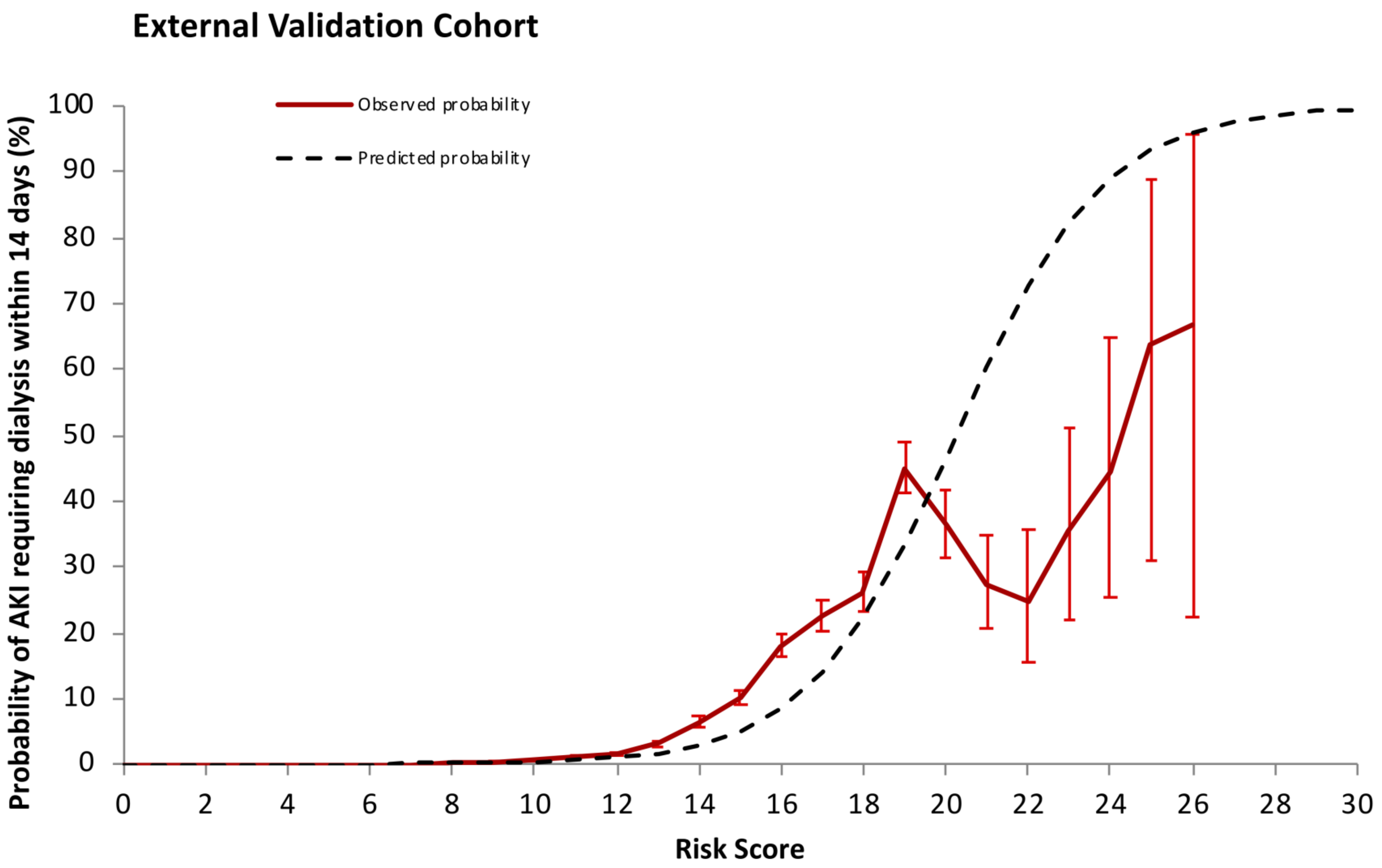

© 2021 Wilson TA et al. *JAMA Network Open*.

eAppendix. Risk Calculator for Acute Kidney Injury Requiring Kidney Replacement Therapy after Non-cardiac Surgery

Risk Calculator for AKI Requiring KRT within 14 days of Non-cardiac Surgery

This Calculator is for adults undergoing non-cardiac surgery

Enter patient's information:

**Age**	63.00	years	**Baseline Hemoglobin**	126	g/L
**Sex**	Male		**Albuminuria**	Not measured	
**Surgery Type**	Vascular		**Myocardial Infarction**	No	
**Baseline Scr**	149	µmol/L	**Liver Disease**	None	

Predicted risk of AKI requiring KRT

Model	Estimated Risk		Predictors
**Model 2 (Alberta)**	**2.76**	**%**	Age, Sex, Surgery Type, Baseline SCr, Baseline Hemoglobin, Albuminuria[¥], MI, Liver Disease
**Model 3 (Alberta)**	**4.88**	**%**	Age, Sex, Surgery Type, Baseline SCr, Baseline Hemoglobin, MI, Liver Disease
**Model 2 (Ontario)**	**3.12**	**%**	Age, Sex, Surgery Type, Baseline SCr, Baseline Hemoglobin, Albuminuria[¥], MI, Liver Disease
**Model 3 (Ontario)**	**6.66**	**%**	Age, Sex, Surgery Type, Baseline SCr, Baseline Hemoglobin, MI, Liver Disease

Variable definitions :

**¥ Albuminuria categories**	Urine Dipstick	Urine ACR (mg/g)	Urine ACR (mg/mmol)
**Normal**	negative	< 30	< 3.4
**Mild**	trace or 1+	30 – 300	3.4 – 33.9
**Heavy**	≥ 2+	> 300	≥ 34

§ Baseline Scr = Pre-operative serum creatinine measurement (1 mg/dL = 88 µmol/L)

Abbreviations: AKI - Acute Kidney Injury, KRT - Kidney Replacement Therapy, Scr - Serum creatinine, MI - Myocardial Infarction, ACR - Albumin to Creatinine Ratio

© 2021 Wilson TA et al. *JAMA Network Open*.
